# Segmentation of medial temporal subregions reveals early right-sided involvement in semantic variant PPA

**DOI:** 10.1186/s13195-019-0489-9

**Published:** 2019-05-10

**Authors:** Martina Bocchetta, Juan Eugenio Iglesias, Lucy L. Russell, Caroline V. Greaves, Charles R. Marshall, Marzia A. Scelsi, David M. Cash, Sebastien Ourselin, Jason D. Warren, Jonathan D. Rohrer

**Affiliations:** 1 Dementia Research Centre, Department of Neurodegenerative Disease, UCL Queen Square Institute of Neurology, University College London, 8-11 Queen Square, London, WC1N 3BG UK; 2 Centre for Medical Image Computing, Department of Medical Physics and Biomedical Engineering, University College London, London, UK; 3 School of Biomedical Engineering and Imaging Sciences, St Thomas’ Hospital, King’s College London, London, UK

**Keywords:** Semantic variant PPA, Magnetic resonance imaging, Medial temporal subregions

## Abstract

**Background:**

Semantic variant of primary progressive aphasia (svPPA) is a subtype of frontotemporal dementia characterized by asymmetric temporal atrophy.

**Methods:**

We investigated the pattern of medial temporal lobe atrophy in 24 svPPA patients compared to 72 controls using novel approaches to segment the hippocampal and amygdalar subregions on MRIs. Based on semantic knowledge scores, we split the svPPA group into 3 subgroups of early, middle and late disease stage.

**Results:**

Early stage: all left amygdalar and hippocampal subregions (except the tail) were affected in svPPA (21–35% smaller than controls), together with the following amygdalar nuclei in the right hemisphere: lateral, accessory basal and superficial (15–23%). On the right, only the temporal pole was affected among the cortical regions. Middle stage: the left hippocampal tail became affected (28%), together with the other amygdalar nuclei (22–26%), and CA4 (15%) on the right, with orbitofrontal cortex and subcortical structures involvement on the left, and more posterior temporal lobe on the right. Late stage: the remaining right hippocampal regions (except the tail) (19–24%) became affected, with more posterior left cortical and right extra-temporal anterior cortical involvement.

**Conclusions:**

With advanced subregions segmentation, it is possible to detect early involvement of the right medial temporal lobe in svPPA that is not detectable by measuring the amygdala or hippocampus as a whole.

**Electronic supplementary material:**

The online version of this article (10.1186/s13195-019-0489-9) contains supplementary material, which is available to authorized users.

## Introduction

Semantic variant of primary progressive aphasia (svPPA) is a subtype of frontotemporal dementia (FTD), characterized clinically by anomia and impaired single-word comprehension. It is associated with a characteristic pattern of asymmetrical antero-inferior temporal lobe atrophy [1–3]. Previous studies of svPPA have shown early left medial temporal lobe involvement, with both hippocampal and amygdalar atrophy [4–6]. However, these studies have investigated the whole hippocampus or amygdala and no previous studies have looked at the subregions of the medial temporal lobe. In this study, we therefore aimed to investigate the pattern of atrophy of the subregions of the hippocampus and the amygdala in svPPA, focusing on the involvement at different stages in order to understand the areas involved early in the disease process.

## Methods

We reviewed the UCL Dementia Research Centre FTD MRI database to identify patients with a diagnosis of svPPA [7] and a usable 3 T T1-weighted magnetic resonance (MR) scan. Twenty-four patients were identified, all with left-temporal predominant disease. Seventy-two cognitively normal subjects with a usable volumetric 3 T T1-weighted MRI were identified as controls. The study was approved by the local ethics committee, and written informed consent was obtained from all participants. The study was conducted in accordance with the Helsinki Declaration of 1975.

Based on their scores on a test of semantic knowledge (the British Picture Vocabulary Scale, BPVS, a word-picture matching task) [8], we split the svPPA patients into three equal subgroups (*n* = 8 per group) of early (BPVS > 110/150), middle (BPVS = 55–110/150) and late disease stage (BPVS < 55/150). Patients were negative for mutations in all FTD-related genes. Two patients received post-mortem confirmation of the underlying neuropathology, both TDP-43 type C.

**Fig. 1:**
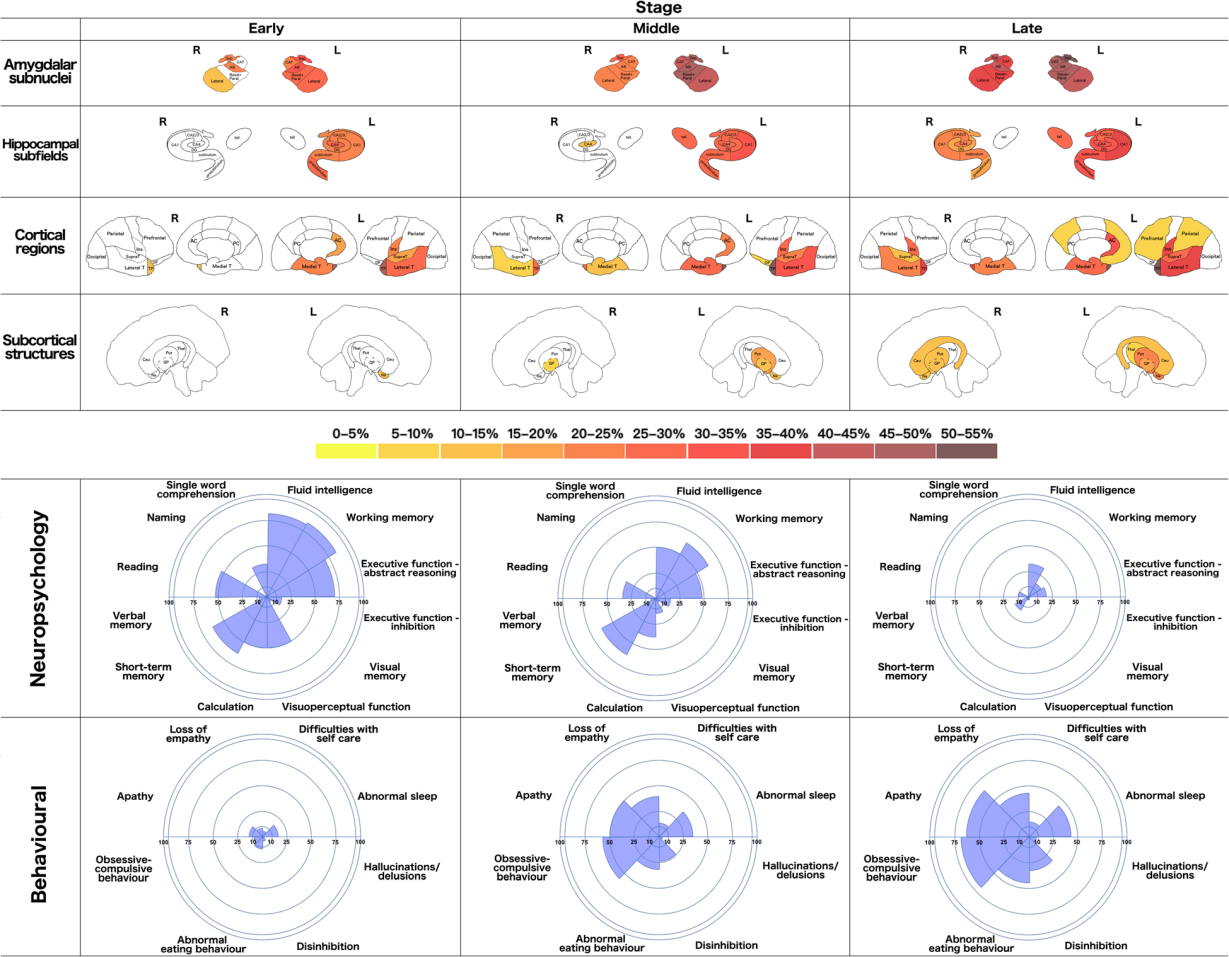
Pattern of atrophy in amygdalar subnuclei, hippocampal subfields, cortical regions and subcortical structures across early, middle and late stages of svPPA. Colour bar denotes the percent difference in volume from controls that remained significant after correction for multiple comparisons. For illustrative purposes, we have included the changes in cognition [mean percentile scores] and behavioural changes [mean percentage score in each Cambridge Behavioural Inventory subscore] that occur at these stages. The length of the segment indicates the severity of the profile. Specifically, for the cognitive performance, the smaller the segment, the worse the performance, whilst for the behavioural symptoms, the bigger the segments, the worse the symptoms

All patients underwent a detailed neuropsychological examination including tests of fluid intelligence (WASI Matrices), single-word comprehension (WASI Vocabulary), naming (Graded Naming Test), reading (National Adult Reading Test), verbal memory (Recognition Memory Test for Words), visual memory (Recognition Memory Test for Faces), short-term memory (forwards digit span), working memory (backwards digit span), calculation (Graded Difficulty Calculation Test), visuoperceptual function (Visual Object and Space Perception battery Object Decision subtest) and executive function (inhibition—D-KEFS Color-Word Ink Naming Test; abstract reasoning—WASI Similarities). A percentile score based on standard norms was generated for each patient, with a mean percentile score created for the early, middle and late stage groups. Assessment of behavioural symptoms was performed using the revised version of the Cambridge Behavioural Inventory (CBI-R) [9]: six subscores were used (difficulties with self-care, abnormal sleep, hallucinations/delusions, disinhibition, abnormal eating behaviour, obsessive-compulsive behaviour, apathy and loss of empathy) with a percentage of the total possible subscore generated for every patient; for each stage, a mean percentage score was created. We report the cognitive and behavioural profiles at each stage for illustrative purposes (Fig. 1 and Additional file 1: Table S1).

T1-weighted MRIs were acquired using a 3-T scanner, either a Trio (Siemens, Erlangen, Germany, TR = 2200 ms, TI = 900 ms, TE = 2.9 ms, acquisition matrix = 256 × 256, spatial resolution = 1.1 mm) or a Prisma (Siemens, Erlangen, Germany, TR = 2000 ms, TI = 850 ms, TE = 2.93 ms, acquisition matrix = 256 × 256, spatial resolution = 1.1 mm). Individuals with moderate to severe vascular disease or space-occupying lesions were excluded.

Volumetric MRI scans were first bias field corrected and whole-brain parcellated using the geodesic information flow (GIF) algorithm [10], which is based on atlas propagation and label fusion. The hippocampal subfields and amygdalar subregions were subsequently segmented using a customized version of the module available in FreeSurfer 6.0 [11, 12], to adapt the output of GIF to the FreeSurfer format. For the hippocampal subfields, we focused on seven areas: CA1, CA2/CA3, CA4, dentate gyrus, subiculum, presubiculum and the tail. We excluded from the analysis the hippocampus-amygdala transition area, the parasubiculum, the molecular layer of the hippocampus, the fimbria and the hippocampal fissure, as they were too small, or not reliably delineated on T1-weighted images. For the amygdalar subnuclei, we focused the analysis on five regions, by combining the smallest subnuclei, based on an anatomical subdivision [13]: lateral nucleus, basal and paralaminar nucleus, accessory basal nucleus, cortico-amygdaloid transition area and the superficial nuclei (central nucleus, cortical nucleus, medial nucleus, anterior amygdaloid area).

For comparison with the medial temporal subregions, we extracted volumes of the following cortical regions from GIF: temporal (medial, lateral, supratemporal, temporal pole), frontal (orbitofrontal, prefrontal), parietal, occipital, insular and cingulate (anterior and posterior). We also extracted volumes of subcortical structures for the pallidum, putamen, caudate, nucleus accumbens and thalamus.

Left and right volumes were corrected for total intracranial volume (TIV), computed with SPM12 v6470 (Statistical Parametric Mapping, Wellcome Trust Centre for Neuroimaging, London, UK) running under Matlab R2014b (Math Works, Natick, MA, USA) [14]. All segmentations were visually checked for quality.

Statistical analyses were performed on brain volumes (as a percentage of TIV) in STATA v14 (Stata-Corp, College Station, TX), between control and patients (early, middle and late stage groups), using a linear regression test adjusting for scanner type, TIV, gender and age. The results were corrected for multiple comparisons (Bonferroni correction): *p* < 0.006 for amygdalar subnuclei and subcortical structures, *p* < 0.005 for hippocampal subfields and *p* < 0.0035 for cortical regions.

## Results

No significant age difference was seen between any of the svPPA groups and controls [Early: 66.9 (5.5) years, Middle: 64.5 (9.5), Late: 64.2 (5.5); Controls: 61.0 (12.1)], *p* = 0.112, *t* test. However, there was a significant difference in gender distribution across stages [Early: 88% male, Middle: 63% male, Late: 25% male; Controls: 40% male], *p* = 0.032, Chi-square test.

Amygdalar subnuclei, hippocampal subfields, cortical regions, subcortical structures, neuropsychology performance and behavioural symptoms at each stage are shown in Fig. 1.

### Early stage

All the left amygdalar and hippocampal subregions (except for the tail) were affected (24–35% and 21–27% smaller than controls, *p* < 0.0005) at this stage, together with the right lateral, accessory basal and superficial nuclei of the amygdala (15–23%, *p* < 0.004) (Table 1).

**Table 1:** Volumetry of amygdalar subnuclei, hippocampal subfields, cortical regions and subcortical structures

			Controls		Early		Middle				Controls		Early		Middle	
	Left								Right							
	Mean	SD	%	*p*-value	%	*p*-value	%	*p*-value	Mean	SD	%	*p*-value	%	*p*-value	%	*p*-value
Amygdalar Subnuclei																
Lateral nucleus																
Controls	0.045	0.005							0.047	0.004						
Early	0.033	0.010	**27**	**< 0.0005**					0.040	0.006	**15**	**0.003**				
Middle	0.026	0.003	**43**	**< 0.0005**	**23**	**< 0.0005**			0.035	0.005	**25**	**< 0.0005**	**12**	**0.005**		
Late	0.025	0.003	**44**	**< 0.0005**	**24**	**< 0.0005**	2	0.723	0.030	0.005	**36**	**< 0.0005**	**25**	**< 0.0005**	14	0.017
Basal and paralaminar nucleus																
Controls	0.033	0.004							0.034	0.003						
Early	0.024	0.006	**29**	**< 0.0005**					0.029	0.006	15	0.012				
Middle	0.018	0.003	**46**	**< 0.0005**	**24**	**< 0.0005**			0.026	0.004	**22**	**< 0.0005**	8	0.092		
Late	0.017	0.002	**48**	**< 0.0005**	**27**	**< 0.0005**	4	0.483	0.021	0.003	**39**	**< 0.0005**	**29**	**< 0.0005**	**22**	**< 0.0005**
Accessory basal nucleus																
Controls	0.018	0.002							0.018	0.002						
Early	0.012	0.004	**32**	**< 0.0005**					0.015	0.004	**21**	**< 0.0005**				
Middle	0.010	0.002	**46**	**< 0.0005**	**20**	**0.002**			0.014	0.002	**24**	**< 0.0005**	4	0.373		
Late	0.009	0.001	**49**	**< 0.0005**	**25**	**< 0.0005**	6	0.482	0.011	0.002	**42**	**< 0.0005**	**27**	**< 0.0005**	**24**	**0.002**
Cortico-amygdaloid transition area																
Controls	0.012	0.002							0.012	0.001						
Early	0.009	0.002	**24**	**< 0.0005**					0.011	0.003	12	0.157				
Middle	0.007	0.001	**44**	**< 0.0005**	**27**	**< 0.0005**			0.009	0.002	**24**	**< 0.0005**	14	0.025		
Late	0.006	0.001	**48**	**< 0.0005**	**32**	**< 0.0005**	7	0.339	0.008	0.002	**36**	**< 0.0005**	**28**	**< 0.0005**	16	0.049
Superficial nuclei (Ce, Co, Me, AAA)																
Controls	0.011	0.002							0.012	0.002						
Early	0.007	0.002	**35**	**< 0.0005**					0.009	0.002	**23**	**0.004**				
Middle	0.006	0.001	**47**	**< 0.0005**	**18**	**0.005**			0.009	0.001	**26**	**< 0.0005**	4	0.341		
Late	0.005	0.001	**51**	**< 0.0005**	**25**	**< 0.0005**	9	0.275	0.007	0.002	**41**	**< 0.0005**	**24**	**0.002**	21	0.024
Hippocampal Subfields																
CA1																
Controls	0.044	0.005							0.047	0.006						
Early	0.035	0.005	**22**	**< 0.0005**					0.045	0.008	5	0.995				
Middle	0.031	0.007	**31**	**< 0.0005**	11	0.020			0.043	0.007	8	0.138	3	0.267		
Late	0.029	0.004	**36**	**< 0.0005**	**18**	**0.001**	7	0.268	0.036	0.006	**24**	**< 0.0005**	**19**	**< 0.0005**	**17**	**0.003**
CA2/CA3																
Controls	0.016	0.002							0.017	0.002						
Early	0.012	0.002	**24**	**< 0.0005**					0.016	0.004	6	0.931				
Middle	0.011	0.002	**27**	**< 0.0005**	3	0.460			0.015	0.003	12	0.064	7	0.184		
Late	0.012	0.002	**26**	**< 0.0005**	2	0.518	−1	0.945	0.013	0.002	**24**	**< 0.0005**	**19**	**0.002**	13	0.054
CA4																
Controls	0.018	0.002							0.019	0.002						
Early	0.013	0.002	**27**	**< 0.0005**					0.017	0.004	**1**0	0.281				
Middle	0.013	0.001	**27**	**< 0.0005**	1	0.342			0.016	0.002	**15**	**0.003**	5	0.156		
Late	0.012	0.001	**34**	**< 0.0005**	9	0.007	9	0.066	0.015	0.002	**21**	**< 0.0005**	**13**	**0.004**	8	0.111
Dentate gyrus																
Controls	0.021	0.002							0.021	0.002						
Early	0.016	0.002	**25**	**< 0.0005**					0.020	0.005	7	0.759				
Middle	0.015	0.002	**27**	**< 0.0005**	4	0.183			0.019	0.003	13	0.021	6	0.132		
Late	0.014	0.002	**32**	**< 0.0005**	10	0.011	6	0.185	0.017	0.003	**19**	**< 0.0005**	**14**	**0.003**	8	0.117
Subiculum																
Controls	0.028	0.003							0.029	0.003						
Early	0.022	0.002	**21**	**< 0.0005**					0.028	0.006	1	0.425				
Middle	0.020	0.003	**28**	**< 0.0005**	10	0.048			0.026	0.005	8	0.116	8	0.074		
Late	0.020	0.004	**31**	**< 0.0005**	**13**	**0.005**	4	0.338	0.022	0.005	**23**	**< 0.0005**	**23**	**< 0.0005**	**16**	**0.004**
Presubiculum																
Controls	0.023	0.003							0.022	0.003						
Early	0.017	0.002	**27**	**< 0.0005**					0.023	0.006	−2	0.173				
Middle	0.016	0.002	**30**	**< 0.0005**	5	0.362			0.021	0.007	5	0.942	6	0.267		
Late	0.016	0.003	**33**	**< 0.0005**	8	0.045	3	0.245	0.018	0.005	**19**	**0.001**	**20**	**0.001**	15	0.015
Hippocampal tail																
Controls	0.041	0.005							0.041	0.005						
Early	0.034	0.006	18	0.019					0.043	0.010	−4	0.055				
Middle	0.030	0.005	**28**	**< 0.0005**	12	0.026			0.042	0.010	−2	0.371	2	0.41		
Late	0.029	0.006	**29**	**< 0.0005**	13	0.009	2	0.624	0.037	0.008	8	0.084	12	0.008	10	0.054
Cortical Regions																
Orbitofrontal																
Controls	0.697	0.047							0.716	0.048						
Early	0.682	0.045	2	0.934					0.727	0.057	**−2**	**0.158**				
Middle	0.629	0.089	**10**	**0.001**	8	0.015			0.716	0.046	**0**	**0.806**	**1**	**0.362**		
Late	0.637	0.063	9	0.009	7	0.062	−1	0.612	0.697	0.078	**3**	**0.647**	**4**	**0.166**	**3**	**0.604**
Prefrontal cortex																
Controls	4.216	0.230							4.322	0.224						
Early	4.087	0.337	3	0.691					4.299	0.379	1	0.545				
Middle	4.045	0.529	4	0.112	1	0.373			4.380	0.369	−1	0.506	−2	0.977		
Late	3.806	0.250	**10**	**0.002**	7	0.047	6	0.245	4.119	0.269	5	0.201	4	0.168	6	0.153
Anterior cingulate																
Controls	0.382	0.039							0.283	0.042						
Early	0.315	0.041	**18**	**0.001**					0.289	0.046	−2	0.339				
Middle	0.300	0.068	**22**	**< 0.0005**	5	0.311			0.318	0.069	−13	0.008	−10	0.204		
Late	0.255	0.026	**33**	**< 0.0005**	**19**	**0.002**	15	0.023	0.288	0.058	−2	0.968	0	0.457	9	0.047
Posterior cingulate																
Controls	0.359	0.038							0.343	0.035						
Early	0.350	0.020	3	0.609					0.368	0.019	−7	0.009				
Middle	0.332	0.025	7	0.065	5	0.320			0.365	0.028	−6	0.022	1	0.747		
Late	0.337	0.028	6	0.169	4	0.535	−1	0.728	0.361	0.047	−5	0.150	2	0.348	1	0.523
Parietal																
Controls	3.224	0.211							3.186	0.229						
Early	3.143	0.229	3	0.538					3.216	0.248	−1	0.049				
Middle	3.147	0.249	2	0.709	0	0.450			3.272	0.200	−3	0.053	−2	0.944		
Late	2.993	0.234	**7**	**0.003**	5	0.008	5	0.046	3.142	0.213	1	0.793	2	0.096	4	0.105
Occipital																
Controls	2.473	0.207							2.564	0.205						
Early	2.393	0.227	3	0.835					2.538	0.195	1	0.575				
Middle	2.395	0.155	3	0.552	0	0.776			2.552	0.175	0	0.697	−1	0.887		
Late	2.432	0.148	2	0.733	−2	0.926	−2	0.853	2.572	0.147	0	0.796	−1	0.817	−1	0.924
Insula																
Controls	0.370	0.035							0.381	0.039						
Early	0.281	0.032	**24**	**< 0.0005**					0.343	0.049	10	0.110				
Middle	0.260	0.036	**30**	**< 0.0005**	7	0.064			0.337	0.038	12	0.007	2	0.425		
Late	0.229	0.021	**38**	**< 0.0005**	**18**	**< 0.0005**	12	0.013	0.267	0.039	**30**	**< 0.0005**	**22**	**< 0.0005**	**21**	**< 0.0005**
Medial temporal																
Controls	1.012	0.062							1.041	0.067						
Early	0.785	0.057	**22**	**< 0.0005**					0.981	0.070	6	0.076				
Middle	0.730	0.056	**28**	**< 0.0005**	7	0.042			0.915	0.070	**12**	**< 0.0005**	7	0.044		
Late	0.743	0.058	**27**	**< 0.0005**	5	0.088	−2	0.787	0.791	0.074	**24**	**< 0.0005**	**19**	**< 0.0005**	**14**	**< 0.0005**
Lateral temporal																
Controls	2.304	0.153							2.345	0.143						
Early	1.652	0.201	**28**	**< 0.0005**					2.231	0.134	5	0.133				
Middle	1.554	0.150	**33**	**< 0.0005**	6	0.084			2.137	0.099	**9**	**< 0.0005**	4	0.105		
Late	1.384	0.159	**40**	**< 0.0005**	**16**	**< 0.0005**	11	0.026	1.864	0.217	**21**	**< 0.0005**	**16**	**< 0.0005**	**13**	**< 0.0005**
Temporal pole																
Controls	0.488	0.056							0.477	0.055						
Early	0.261	0.066	**47**	**< 0.0005**					0.413	0.071	**13**	**0.006**				
Middle	0.231	0.035	**53**	**< 0.0005**	12	0.187			0.352	0.049	**26**	**< 0.0005**	15	0.019		
Late	0.228	0.029	**53**	**< 0.0005**	13	0.324	1	0.766	0.287	0.038	**40**	**< 0.0005**	**30**	**< 0.0005**	18	0.048
Supratemporal																
Controls	0.430	0.050							0.369	0.039						
Early	0.348	0.037	**19**	**< 0.0005**					0.357	0.045	3	0.910				
Middle	0.336	0.046	**22**	**< 0.0005**	4	0.359			0.368	0.040	0	0.718	−3	0.855		
Late	0.301	0.056	**30**	**<0.0005**	14	0.017	10	0.122	0.322	0.054	**13**	**0.004**	10	0.028	12	0.016
Subcortical Structures																
Nucleus accumbens																
Controls	0.040	0.003							0.038	0.003						
Early	0.035	0.003	**13**	**<0.0005**					0.035	0.003	9	0.048				
Middle	0.034	0.005	**15**	**<0.0005**	3	0.235			0.036	0.004	5	0.155	−4	0.638		
Late	0.030	0.003	**24**	**<0.0005**	**13**	**0.001**	10	0.019	0.032	0.004	**15**	**<0.0005**	7	0.026	11	0.007
Caudate																
Controls	0.237	0.026							0.248	0.024						
Early	0.221	0.020	7	0.508					0.235	0.026	5	0.598				
Middle	0.222	0.026	6	0.350	0	0.851			0.237	0.024	4	0.507	−1	0.929		
Late	0.207	0.030	**12**	**0.001**	6	0.037	7	0.053	0.217	0.036	**12**	**0.001**	8	0.044	8	0.050
Pallidum																
Controls	0.129	0.014							0.130	0.013						
Early	0.114	0.007	12	0.010					0.119	0.007	8	0.123				
Middle	0.113	0.008	**12**	**<0.0005**	1	0.160			0.119	0.008	8	0.004	0	0.303		
Late	0.104	0.009	**19**	**<0.0005**	9	0.016	8	0.270	0.111	0.011	**14**	**<0.0005**	6	0.054	7	0.336
Putamen																
Controls	0.307	0.031							0.305	0.031						
Early	0.268	0.018	13	0.011					0.289	0.016	5	0.981				
Middle	0.255	0.023	**17**	**<0.0005**	5	0.044			0.277	0.023	9	0.019	4	0.081		
Late	0.237	0.018	**23**	**<0.0005**	**11**	**0.001**	7	0.144	0.261	0.022	**14**	**<0.0005**	**10**	**0.002**	6	0.142
Thalamus																
Controls	0.400	0.035							0.392	0.039						
Early	0.357	0.024	11	0.024					0.380	0.032	3	0.279				
Middle	0.362	0.029	9	0.008	−2	0.791			0.387	0.036	1	0.258	−2	0.992		
Late	0.364	0.027	**9**	**<0.0005**	−2	0.169	−1	0.255	0.388	0.027	1	0.226	−2	0.093	0	0.086

Values denote mean and standard deviation (SD) volumes as the percentage of the total intracranial volume (TIV) or difference (%). *p* values denote significance on linear regression test. Bold represents a significant difference between the groups after correcting for multiple comparisons

Outside of the medial temporal lobe, on the left, all the temporal cortical regions (19–47%, *p* < 0.0005) were affected as well as the anterior cingulate (18%, *p* = 0.001) and insula (24%, *p* < 0.0005). The left nucleus accumbens was the only other subcortical structure affected (13%, *p* < 0.0005). Apart from the affected amygdalar subnuclei, the only other right hemisphere structure affected at this stage was the temporal pole (13%, *p* = 0.006).

Cognitively, patients showed severely impaired naming already, with relatively preserved working memory, abstract reasoning and fluid intelligence. Behavioural symptoms were mild and mainly related to abnormal eating behaviour, apathy and abnormal sleep.

### Middle stage

At this stage, the left hippocampal tail became affected (28%, *p* < 0.0005), together with the other right amygdalar nuclei (22–26%, *p* < 0.0005) and the right CA4 region of the hippocampus (15%, *p* = 0.003).

Cortically, the left orbitofrontal lobe was affected at this stage along with more posterior temporal structures on the right: lateral and medial temporal cortices (9–12%, *p* < 0.0005). Subcortically, the left pallidum and putamen were affected (12–17%, *p* < 0.0005) and the right pallidum (8%).

Cognitively, single-word comprehension and reading became increasingly impaired, but working memory, short-term memory and abstract reasoning remained relatively intact. Behavioural symptoms increased with the presence of obsessive-compulsive behaviour and loss of empathy as well as abnormal eating behaviour, apathy and disinhibition.

### Late stage

In the late stage, the remaining right hippocampal regions (except the tail) (19–24%, *p* < 0.001) became affected.

Cortically, spread to the left prefrontal and parietal cortices was seen whilst on the right, the insula (30%) and supratemporal cortex (13%, *p* < 0.004) were affected. Subcortically, the left caudate, thalamus and right nucleus accumbens, caudate and putamen were affected (12–15%).

At this stage, all cognitive domains were severely impaired except for short-term and working memory, abstract reasoning and fluid intelligence. Severe behavioural symptoms were seen.

## Discussion

Using advanced subregional segmentation, we were able to detect early involvement in the right hemisphere in svPPA, with progression of atrophy through the medial temporal lobes as the disease moves from early to middle to late stage.

Extensive medial temporal atrophy is seen on the left in most amygdalar and hippocampal subregions at the earliest stage of svPPA, co-incidental with the involvement of all of the temporal cortices on the left. This is consistent with previous studies showing that even at first clinical presentation, significant left temporal lobe atrophy is present [1, 15].

Previous studies have not shown early involvement of the right medial temporal structures. In this study, the earliest subnuclei affected on the right were the accessory basal, lateral and superficial nuclei of the amygdala. These subnuclei are interconnected and receive input from the temporal pole and the hippocampus (also affected on the right in the early stage) as well as other parts of the temporal and frontal cortices and the nucleus accumbens [13, 16]. The ability to use advanced subregional segmentation techniques in this study allows early detection of right medial temporal atrophy.

The cognitive and behavioural correlates of the individual right amygdalar subnuclei are poorly studied, but prior studies of the whole amygdala implicate the right side as being important in the processing of emotional information [17, 18]. In our study, loss of empathy is mildly affected at the earliest stage (Fig. 1): this is likely to represent an impairment of self-knowledge, a process that requires the linking of emotions with semantics, and has previously been shown to be associated with right temporal lobe atrophy including the amygdala [19]. The particular amygdalar subnuclei affected early are part of the limbic network and therefore likely to be intrinsically involved in emotion processing [16].

Of all the medial temporal subregions, the hippocampal tail is preserved until the later stages of svPPA. This is in line with previous studies, where the posterior temporal lobe is spared and an antero-posterior gradient is present [20, 21]. Indeed, svPPA patients typically show intact episodic memory and spatial navigation, functions typically linked to the hippocampal tail. Consistent with the theory of svPPA as a network-opathy [22], the first hippocampal region to become affected on the right is CA4, an area highly connected to the temporal cortex and amygdala [23].

Limitations of the study include using cross-sectional data with staging of the disease by impairment on a task of semantic knowledge and the small number of svPPA cases. Further studies would benefit from the analysis of longitudinal data from a larger sample to see whether the same pattern is seen. Despite the gold standard still being manual segmentation of dedicated MRIs or on brain tissue post-mortem, these automated methods included in this study have been previously validated and proven reliable to delineate the subregions on T1-MRI (Dice coefficients > 0.86; ICC 0.88–0.93) [10–12, 24, 25]. Moreover, in this study, we carefully excluded small subregions and combined together groups of nuclei to improve the anatomical validity. Automated segmentations will play a key role in the future, as manual segmentations are likely to be unfeasible for large cohorts of patients.

## Additional file

Additional file 1: **Table S1.** Cognitive and behavioural variables for the svPPA patients. *p* values denote significance on Kruskal-Wallis test among the three groups. (DOCX 17 kb)

## References

[1] Rohrer JD, Warren JD, Modat M, Ridgway GR, Douiri A, Rossor MN, Ourselin S, Fox NC (2009). Patterns of cortical thinning in the language variants of frontotemporal lobar degeneration. Neurology..

[2] Rohrer JD, Rosen HJ (2013). Neuroimaging in frontotemporal dementia. Int Rev Psychiatry.

[3] Schroeter ML, Raczka K, Neumann J, Yves von Cramon D (2007). Towards a nosology for frontotemporal lobar degenerations-a meta-analysis involving 267 subjects. Neuroimage..

[4] Rohrer JD, McNaught E, Foster J, Clegg SL, Barnes J, Omar R, Warrington EK, Rossor MN, Warren JD, Fox NC (2008). Tracking progression in frontotemporal lobar degeneration: serial MRI in semantic dementia. Neurology..

[5] Lehmann M, Douiri A, Kim LG, Modat M, Chan D, Ourselin S, Barnes J, Fox NC (2010). Atrophy patterns in Alzheimer’s disease and semantic dementia: a comparison of FreeSurfer and manual volumetric measurements. Neuroimage..

[6] Nestor PJ, Fryer TD, Hodges JR (2006). Declarative memory impairments in Alzheimer’s disease and semantic dementia. Neuroimage..

[7] Gorno-Tempini ML, Hillis AE, Weintraub S, Kertesz A, Mendez M, Cappa SF, Ogar JM, Rohrer JD, Black S, Boeve BF, Manes F, Dronkers NF, Vandenberghe R, Rascovsky K, Patterson K, Miller BL, Knopman DS, Hodges JR, Mesulam MM, Grossman M (2011). Classification of primary progressive aphasia and its variants. Neurology..

[8] Dunn DM, Dunn LM, National Foundation for Educational Research in England and Wales, GL Assessment (Firm). 2009. 3rd ed. GL Assessment. ISBN-10: 0708719554.

[9] Wear HJ, Wedderburn CJ, Mioshi E, Williams-Gray CH, Mason SL, Barker RA, Hodges JR (2008). The Cambridge Behavioural Inventory revised. Dement Neuropsychol.

[10] Cardoso M. Jorge, Modat Marc, Wolz Robin, Melbourne Andrew, Cash David, Rueckert Daniel, Ourselin Sebastien (2015). Geodesic Information Flows: Spatially-Variant Graphs and Their Application to Segmentation and Fusion. IEEE Transactions on Medical Imaging.

[11] Saygin ZM, Kliemann D, Iglesias JE, van der Kouwe AJW, Boyd E, Reuter M, Stevens A, Van Leemput K, McKee A, Frosch MP, Fischl B, Augustinack JC, Alzheimer’s Disease Neuroimaging Initiative (2017). High-resolution magnetic resonance imaging reveals nuclei of the human amygdala: manual segmentation to automatic atlas. Neuroimage..

[12] Iglesias JE, Augustinack JC, Nguyen K, Player CM, Player A, Wright M, Roy N, Frosch MP, McKee AC, Wald LL, Fischl B, Van Leemput K (2015). A computational atlas of the hippocampal formation using ex vivo, ultra-high resolution MRI: application to adaptive segmentation of in vivo MRI. Neuroimage..

[13] deCampo DM, Fudge JL (2012). Where and what is the paralaminar nucleus? A review on a unique and frequently overlooked area of the primate amygdala. Neurosci Biobehav Rev.

[14] Malone IB, Leung KK, Clegg S, Barnes J, Whitwell JL, Ashburner J, Fox NC, Ridgway GR (2015). Accurate automatic estimation of total intracranial volume: a nuisance variable with less nuisance. Neuroimage..

[15] Czarnecki K, Duffy JR, Nehl CR, Cross SA, Molano JR, Jack CR, Shiung MM, Josephs KA, Boeve BF (2008). Very early semantic dementia with progressive temporal lobe atrophy: an 8-year longitudinal study. Arch Neurol.

[16] LeDoux J (2007). The amygdala. Curr Biol.

[17] Rosen HJ, Perry RJ, Murphy J, Kramer JH, Mychack P, Schuff N, Weiner M, Levenson RW, Miller BL (2002). Emotion comprehension in the temporal variant of frontotemporal dementia. Brain..

[18] Snowden Julie S., Harris Jennifer M., Thompson Jennifer C., Kobylecki Christopher, Jones Matthew, Richardson Anna M., Neary David (2018). Semantic dementia and the left and right temporal lobes. Cortex.

[19] Sollberger M, Rosen HJ, Shany-Ur T, Ullah J, Stanley CM, Laluz V, Weiner MW, Wilson SM, Miller BL, Rankin KP (2014). Neural substrates of socioemotional self-awareness in neurodegenerative disease. Brain Behav.

[20] La Joie R, Perrotin A, de La Sayette V, Egret S, Doeuvre L, Belliard S, Eustache F, Desgranges B, Chételat G (2013). Hippocampal subfield volumetry in mild cognitive impairment, Alzheimer’s disease and semantic dementia. Neuroimage Clin.

[21] Tan RH, Wong S, Kril JJ, Piguet O, Hornberger M, Hodges JR, Halliday GM (2014). Beyond the temporal pole: limbic memory circuit in the semantic variant of primary progressive aphasia. Brain..

[22] Fletcher PD, Warren JD (2011). Semantic dementia: a specific network-opathy. J Mol Neurosci.

[23] de Flores R, Mutlu J, Bejanin A, Gonneaud J, Landeau B, Tomadesso C, Mézenge F, de La Sayette V, Eustache F, Chételat G (2017). Intrinsic connectivity of hippocampal subfields in normal elderly and mild cognitive impairment patients. Hum Brain Mapp.

[24] Herten Annika, Konrad Kerstin, Krinzinger Helga, Seitz Jochen, von Polier Georg G. (2018). Accuracy and bias of automatic hippocampal segmentation in children and adolescents. Brain Structure and Function.

[25] Whelan CD, Hibar DP, van Velzen LS, Zannas AS, Carrillo-Roa T, McMahon K, Prasad G, Kelly S, Faskowitz J, deZubiracay G, Iglesias JE, van Erp TGM, Frodl T, Martin NG, Wright MJ, Jahanshad N, Schmaal L, Sämann PG, Thompson PM, Alzheimer’s Disease Neuroimaging Initiative (2016). Heritability and reliability of automatically segmented human hippocampal formation subregions. Neuroimage..

