# Supplementary material for: Segmentation of medial temporal subregions reveals early right-sided involvement in semantic variant PPA
Source: Alzheimers Res Ther. 2019 May 10;11:41. doi: 10.1186/s13195-019-0489-9 (PMC6511178; doi:10.1186/s13195-019-0489-9)
Supplement: Supplementary file 1 — Table S1. Cognitive and behavioural variables for the svPPA patients. p values denote significance on Kruskal-Wallis test among the three groups. (DOCX 17 kb) [file 13195_2019_489_MOESM1_ESM.docx]

**Additional file 1: Table S1**

Cognitive and behavioural variables for the svPPA patients. P-values denote significance on Kruskal-Wallis test among the three groups.

|  |  | **WASI-m** | **WASI-v** | **GNT** | **NART** | **RMT-w** | **RMT-f** | **FDS** | **BDS** | **GDC** | **VOSP** | **Stroop** | **WASI-s** | **Self-care** | **Sleep** | **HD** | **Disinh** | **Eat** | **OCb** | **Apathy** | **Empathy** |
| --- | --- | --- | --- | --- | --- | --- | --- | --- | --- | --- | --- | --- | --- | --- | --- | --- | --- | --- | --- | --- | --- |
| **Early** | **Mean** | 26.9 | 54.4 | 5.4 | 26.1 | 39.4 | 35.6 | 8.9 | 8.0 | 17.5 | 18.8 | 72.5 | 27.9 | 0.9 | 1.3 | 0.1 | 1.0 | 1.9 | 1.3 | 1.1 | 1.0 |
|  | **SD** | 3.0 | 10.0 | 6.6 | 12.1 | 6.2 | 7.5 | 2.3 | 2.6 | 4.6 | 0.7 | 41.3 | 8.3 | 2.3 | 1.7 | 0.4 | 0.8 | 2.0 | 0.8 | 2.3 | 0.8 |
| **Middle** | **Mean** | 21.9 | 27.6 | 0.1 | 19.7 | 33.1 | 32.4 | 9.5 | 7.5 | 9.3 | 17.0 | 74.3 | 17.9 | 2.1 | 2.7 | 0.4 | 5.7 | 5.1 | 9.1 | 4.0 | 4.7 |
|  | **SD** | 7 | 18 | 0 | 14 | 10 | 7 | 2 | 3 | 6 | 2 | 28 | 8 | 4 | 2 | 1 | 8 | 5 | 5 | 3 | 4 |
| **Late** | **Mean** | 18.6 | 3.8 | 0.0 | 9.9 | 28.7 | 30.0 | 5.8 | 4.0 | 2.5 | 13.1 | 100.3 | 4.9 | 2.4 | 3.4 | 0.9 | 7.9 | 7.1 | 11.0 | 5.1 | 5.0 |
|  | **SD** | 7 | 4 | 0 | 10 | 4 | 6 | 3 | 2 | 3 | 5 | 21 | 2 | 4 | 3 | 1 | 6 | 4 | 5 | 3 | 3 |
| p-value | **Kruskal-Wallis** | 0.018 | <0.0005 | 0.001 | 0.042 | 0.056 | 0.403 | 0.042 | 0.021 | 0.001 | 0.003 | 0.164 | <0.0005 | 0.470 | 0.143 | 0.253 | 0.026 | 0.034 | 0.001 | 0.056 | 0.015 |

Abbreviations:

WASI-m: WASI Matrices (maximum 32); WASI-v: WASI Vocabulary (maximum 80); GNT: Graded Naming Test (maximum 30); NART: National Adult Reading Test (maximum 50); RMT-w: Recognition Memory Test for Words (maximum 50); RMT-f: Recognition Memory Test for Faces (maximum 50); FDS: Forwards digit span (maximum 12); BDS: Backwards digit span (maximum 12); GDC: Graded Difficulty Calculation Test (maximum 24); VOSP: Visual Object and Space Perception battery Object Decision subtest (maximum 20); Stroop: inhibition – D-KEFS Color-Word Ink Naming Test (time in seconds); WASI-s: WASI Similarities (maximum 48); Behavioural scores from the Cambridge Behavioural Inventory – Revised: Self-care: Difficulties with self care (maximum 16); Sleep: Abnormal sleep (maximum 8); HD: Hallucinations/delusions (maximum 12); Disinh: Disinhibition (maximum 24); Eat: Abnormal eating behaviour (maximum 16); OCb: Obsessive-compulsive behaviour (maximum 16); Apathy (maximum 8); Empathy: Loss of empathy (maximum 12).
